# Explanations for variations in hospital expenditures among four large California counties

**DOI:** 10.1186/s12913-023-09390-y

**Published:** 2023-04-22

**Authors:** Aimee J. Lansdale, Robert M. Kaplan

**Affiliations:** 1 Stanford Prevention Research Center, Stanford University School of Medicine, Stanford, USA; 2 Department of Epidemiology, University of California, San Francisco, USA; 3 Clinical Excellence Research Center, Stanford University School of Medicine, Stanford, USA

**Keywords:** Small area variation, Payment models, Competition, Medicare data, California

## Abstract

**Objective:**

To investigate competing explanations for why Medicare Fee for Service (FFS) and private sector payments lead to hospital cost variations in Californian counties.

**Data sources:**

Ratios of private to Medicare hospital costs were obtained from state-based all-payer claims databases. Demographics were estimated from the U.S. Census Bureau and the California Health Interview Survey. Medicaid and Medicare spending was obtained from Kaiser Family Foundation. Medicare Advantage enrollment was obtained from the California Department of Health Care Services and market consolidation was estimated using the Herfindahl–Hirschman Index (HHI).

**Study design:**

Per capita costs, demographics, Medicaid and Medicare spending, Medicare Advantage enrollment, and HHI scores were compared for San Francisco (SF), Sacramento, Los Angeles (LA), and San Diego (SD).

**Principal findings:**

LA hospitals had the lowest per capita private insurer costs, but the highest Medicare FFS costs. The findings might be explained by a lower HHI for LA, indicating a more competitive market, than SD, SF, and Sacramento.

**Conclusions:**

Medicare FFS hospital costs do not provide an accurate representation of health care spending in Californian counties. In more competitive markets, private insurance companies can negotiate lower prices, while oversupply may allow facilities to increase volume in Medicare FFS.

## Introduction

In 2020, the United States spent about $4.1 trillion or $12,530 per person on health care [1]. At approximately 19.7% of its Gross Domestic Product (GDP), the US had the highest percentage among the Organization for Economic Co-operation and Development countries [1]. As the number of individuals 85 years and older is expected to grow nearly 200 percent by 2060, continued growth in health care expenditures are expected [2]. In addition, the growing number of individuals with pre-diabetes, diabetes, and obesity may increase health care spending. About 44.1 million individuals are projected to have diabetes with annual spending related to diabetes alone expected to be $336 billion in 2034 (in constant 2007 USD) [3]. Between 2012 and 2017, the economic costs of diabetes increased by 26% [4]. Recent estimates suggest that the number of adults with diabetes was 22.3 million (9.1%) in 2014, but will increase to 39.7 million (13.9%) by 2030 [5]. Despite the increase in diabetes and obesity, costs of health care continue to differ substantially in defined geographic regions, even among communities with comparable disease burden.

When examining the population in the United States by insurance groups, Medicaid and Medicare users only make up 19.8% and 14.2% of the population (data from CMS,gov), respectively [6]. Both estimates include about 12.5 million people who are enrolled in both programs. In 2019, about 17% of the Medicare population received Medicaid and about 14% of the Medicaid population also got Medicare benefits [7]. Almost half of the country’s population, about 49.6%, have private insurance policies from employers [6]. However, most policy analyses concentrate on the minority who are insured by government programs. Publicly available data on individuals with private insurance policies has been limited. As a result, health care policymakers use data from the Center for Medicare and Medicaid Services (CMS) to make recommendations for the entire population, even individuals with private insurance policies. Although this is changing as claims data for employer-sponsored health care plans become available, it is still the norm to use databases on Medicare and Medicaid data such as the Dartmouth Atlas of Healthcare [8]. The Atlas has documented variations in the distribution and use of medical resources using Medicare and Medicaid data for over 20 years and has become the gold standard for US health care data.

Recent analyses highlight substantial differences in payment between public and private insurers. Schulman and Milstein showed that US Medicare and Medicaid payments to hospital were, on average lower than the hospital costs [9]. Conversely, private insurers were paying at rates that were more than 140% of hospital costs [10]. In other words, private insurers subsidize public payers. These private insurers pay between 2 and 5 times the rate paid by Medicare and Medicaid.

In this paper, we compare healthcare costs in the four major metropolitan health care markets in California. We focus on this single large state because California has a population larger than most countries (nearly 40 million people), is ethnically and racially diverse, and has environmental variability. Limiting the analysis to one state reduces the extraneous influence of different Medicaid policies across states and the influence of variability in insurance regulatory rules.

## Methods

Data for this analysis came from several sources. To estimate hospital prices, we used data on self-insured employers, state-based all-payer claims databases, and health plans that have been assembled by RAND [11]. The hospital data were from 2016–2018. However, we believe these indicators remain relatively stable. Relative prices were estimated from the allowed amount paid by the private plan as a percentage of what Medicare pays (by pre-negotiated contract) the same hospital for the same services.

Hospital price data were categorized by county group based on zip code. Statistical tests and descriptive analyses, and graphic displays were completed using R. The U.S. Census Bureau 2020 [12] and the California Health Interview Survey (CHIS) [13] were used to better understand the demographic makeup of each county. Other cost data from Kaiser Family Foundation (KFF) [6] were used to calculate 2019 Medicaid and Medicare spending per enrollee.

Data from the California Department of Health Care Services were used to examine Medicare Advantage enrollment data by county [14]. State- and county-level enrollment numbers are from August 2021. To examine how competitive the different health care markets are and the concentration of hospitals in each of the various markets, we used the Herfindahl–Hirschman Index (HHI) from the Yale Tobin Center for Economic Policy [15].

A general linear model analysis created an identifier for each county by aggregating zip codes falling within county geographic boundaries. Each county was then contrasted with the remaining zip codes in the state.

## Results

**Fig. 1:**
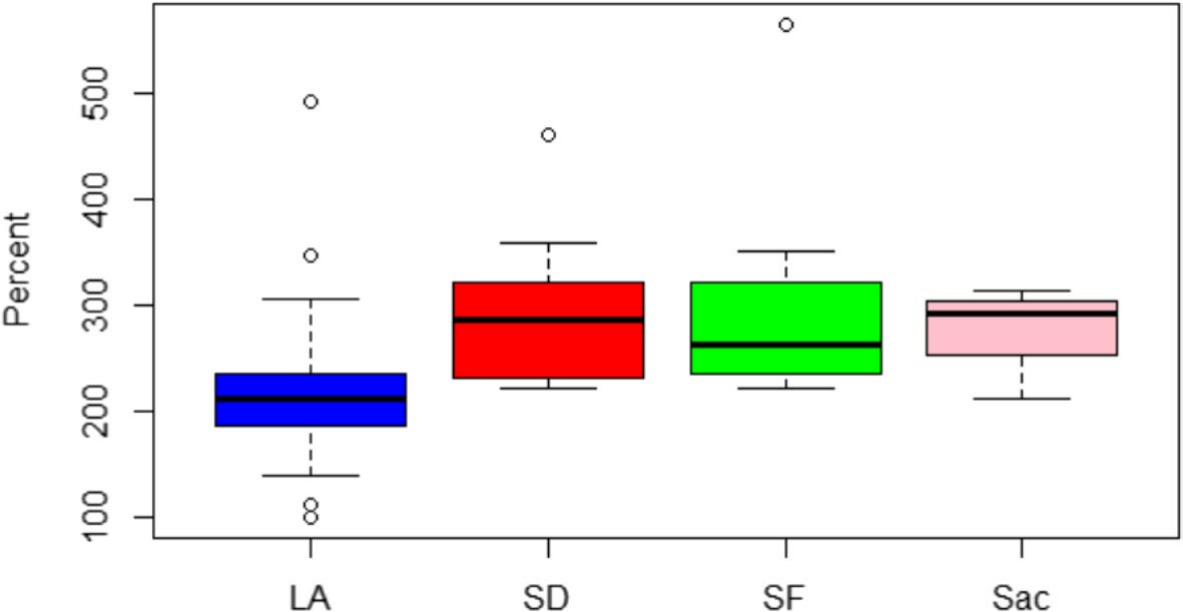
Relative price of allowed amount by private plans as a percentage of what Medicare would have paid for inpatient and outpatient services [11]

Interactive data maps on the Dartmouth Atlas website show price-adjusted Medicare reimbursements per enrollee were higher in LA ($14,361.41) in comparison to San Diego ($10,966.41), San Francisco ($9,214.26), and Sacramento ($9,494.30) counties [16]. The RAND data includes hospital price information from self-insured employers, state-based all-payer claims databases, and health plans. Prices reflect the negotiated allowed amount paid per service, including amounts from the health plan and the patient, with adjustments for the intensity of services provided. We specifically examined what private insurers pay hospitals in relation to Medicare contracted rates for the relative price for inpatient and outpatient services. We expected LA to have a similar or higher ratio when comparing the amount private insurers pay hospitals in relation to Medicare, as seen in the Dartmouth Atlas data. The analysis revealed that the ratio was lower in LA (2.15) than San Diego (2.93), San Francisco (3.00), and Sacramento (2.77) counties (Fig. 1). The general linear model analysis demonstrated that the ratio of private payer to Medicare payments for inpatient and outpatient services was significantly lower in Los Angeles in comparison to the rest of California (95% CI 1.26–93.1, *p* = 0.04), but was non-significant for the other three counties.

We considered four alternative explanations for why the ratio of what private insurers pay hospitals in relation to Medicare for the relative price for inpatient and outpatient services is lower in LA (2.15) than San Diego (2.93), San Francisco (3.00), and Sacramento (2.77) counties. The hypotheses included:

1. LA, San Diego, San Francisco, and Sacramento counties each serve different demographic populations (age, race, income, education, percent uninsured, etc.). Is it possible demographic differences explain why the ratio of what private insurers pay hospitals is lower in LA?
2. The Dartmouth Atlas Project data only considers Medicare FFS costs. However, Medicare Advantage (MA) attracts somewhat lower cost seniors. As a result, the FFS program is left with more expensive seniors. Counties with high MA enrollment may appear to be more expensive simply because they have a greater concentration of FFS enrollees.
3. Medicare is costlier in southern California. However, the opposite is true for Medicaid.
4. The more competitive the health care market and the higher concentration of hospitals in a market, the less hospitals can charge private insurance companies.

Not only is the population in LA is approximately three times as large as the population in San Diego, 6 times as large as Sacramento, and 11.5 times as large as San Francisco, but the demographic composition of LA is distinct. In comparison to the other counties, LA has a higher percentage of Hispanics and a lower percentage of white, non-Hispanic residents (Table 1).

**Table 1:** Comparison of Los Angeles, San Diego, Sacramento, and San Francisco Demographic Profiles, 2020

County	Los Angeles	San Diego	Sacramento	San Francisco
Geographic area (square miles)	4,058.2	4,208.0	964.9	46.9
Population (n)	10,014,009	3,298,634	1,585,055	873,965
Percent Hispanic^a^	48.0	33.8	23.6	15.6
Percent white, Non-Hispanic^a^	25.6	43.1	41.0	39.1
Percent Black^a^	7.9	4.7	9.6	5.3
Percent Asian-Pacific Islander^a^	15.2	12.9	19.0	34.3
Percent high school or equivalent degree	20.4	18.2	22.1	11.6
Percent bachelor’s degree or higher	33.5	39.5	31.4	58.8
Median age (years)	36.7	36.1	36.4	38.3
Persons 65 years or older (percent)	13.6	14.1	14.1	15.8
Median household income	$71,358	$82,426	$70,684	$119,136
Persons below poverty (percent)	14.2	10.9	13.9	10.1
Disability Persons with disability (percent)	10.1	10.0	12.0	10.1
Percentage without health care coverage	9.2	7.6	5.5	3.6

Data from U.S. Census Bureau

^a^Percentages for the race categories do not add to 100% because some categories were not included

Considering differences in education, Los Angeles had a similar percentage of individuals with only a high school or an equivalent degree as San Diego and Sacramento counties and a lower percentage than San Francisco. These differences in educational attainment education are associated with income differentials. Median income in LA is $71,358, which is much lower than the median income of San Francisco and San Diego, $119,136 and $82,426, respectively. LA also has the highest percentage of persons below poverty at 14.2%. LA has a higher percentage of individuals without health insurance at 9.2% compared to San Diego at 7.6%, Sacramento at 5.5%, and San Francisco at 3.6%.

### Hypothesis 1

Health care costs are known to vary by the demographic composition of the local population. CHIS data showed the burden of disease is significantly higher in LA with only 56.9% having very good or excellent health status and 14.1% of the population having poor and fair self-rated health status (San Francisco: 67.5% very good/excellent and 9.5% fair/poor; Sacramento: 60.1% very good/excellent and 9.5% fair/poor; San Diego: 67.4% very good/excellent and 9.1 fair/poor) [13]. In addition, when uninsured individuals require health care, hospitals and health providers are often not able to recover fees for their services. To make up for these costs, hospitals and health providers increase the costs of those who can pay, such as individuals with private insurance. As a result, the higher the uninsured rate in the population, the higher medical charges for all other individuals living in that area.

LA’s uninsured rate is the highest at 7.9% (or 784,000 individuals) among the four comparison counties (San Diego: 7.2%, Sacramento: 5.5%, and San Francisco: 2.8%) [13]. Since the population in LA has lower self-rated health and a higher proportion of residents from traditionally underserved demographic groups compared to San Francisco, Sacramento, and San Diego, if this hypothesis were correct, one would expect LA to have the highest relative price for inpatient and outpatient services. To support this hypothesis, LA would be expected to have the highest relative price for inpatient and outpatient services. However, this hypothesis was not supported by the RAND data, which suggest LA has the lowest relative price for both inpatient and outpatient services.

### Hypothesis 2

Medicare Advantage is believed to appeal to healthier seniors, while FFS is thought to be more attractive to those expected to need more specialized services. This may remove the healthier and lower cost seniors from the traditional FFS Medicare program because they are less sick. As a result, the FFS program is left with more expensive seniors. The Dartmouth Atlas Health Care data only consider FFS costs in their analysis. If this hypothesis were true, MA would be higher in LA. However, enrollment in MA is not necessarily higher in southern California counties compared to northern California counties. For example, as shown in Table 2, Sacramento County has a MA enrollment of 45.5% [14].

**Table 2:** Medicare Advantage (MA) Penetration by County, March 2021 14

**County**	**MA, Among Dual Beneficiaries (and % of County Dual Beneficiaries)**^a^	
Los Angeles	219,230	49.0%
San Diego	53,797	57.4%
San Francisco	13,170	28.2%
Sacramento	26,617	45.5%

^a^Dual beneficiaries refers to dual Medicare/Medi-Cal beneficiaries

### Hypothesis 3

**Fig. 2:**
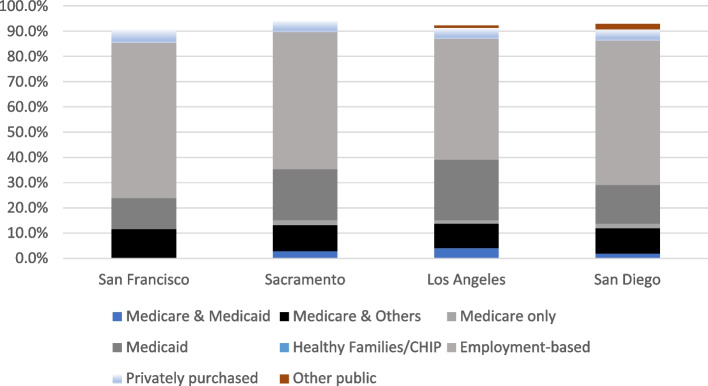
Type of current health insurance coverage—all ages. Source: Data from California Health Interview Survey 2019. UCLA Center for Health Policy Research

Using the number of individuals enrolled in Medicare (including Medicare Advantage) Medicaid, dual eligible, Children’s Health Insurance Program (CHIP), Employer-based, privately purchased, and other public, we estimated the penetrance of insurance type by county. When examining the counties by current health insurance coverage, LA had the highest percentage of individuals enrolled in Medicaid and the lowest percentage of individuals enrolled in Medicare (only). In comparison to Los Angeles (dual eligible = 4.1% CI 3.4%-4.9%) there were significantly fewer high-cost dual eligible patients in Sacramento (2.9%) and San Diego (2.0%). San Francisco had a higher rate (4.8%) although the difference was not statistically significant (see Fig. 2).

**Fig. 3:**
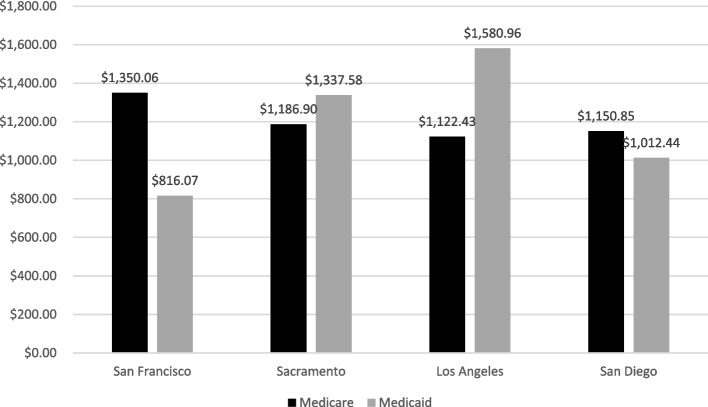
Medicare vs. Medicaid costs in 2019. Source: UCLA Center for Health Policy Research & Kaiser Family Foundation

If the hypothesis that in California, Medicare is costliest and Medicaid is least costly in southern California were true, one would expect lower costs in LA. However, this was not the result. By multiplying the number of individuals enrolled in Medicare and Medicaid in each county from the CHIS data [13] by Medicare and Medicaid spending per enrollee [6], we calculated the costs of each insurance type by county in 2019. Medicare was defined as Medicare plus others (Medicare beneficiaries who report a non-HMO (e.g., EPO, PPO) Medicare Advantage plan). Contrary to expectation, Medicare costs were lowest in LA in comparison to the other counties (Fig. 3). While the costs for Medicaid are in fact higher in LA than San Diego, San Francisco, and Sacramento.

### Hypothesis 4

The Herfindahl–Hirschman Index (HHI) quantifies market competition through concentration of hospitals in each county [15]. LA had the lowest HHI (1,136), which indicates it has the most competitive market (San Diego: 2,584, Sacramento: 2,887, and San Francisco: 2,039) [15]. In competitive markets, with an oversupply of hospitals, insurance companies have more leverage to negotiate lower prices. Contrastingly, when hospitals do not compete on price, such as on Medicare FFS, hospitals can use their excess capacity to increase volume. However, the highly competitive market in LA may have resulted in lower charges to private insurers. This reasoning could explain why the ratio of what private insurers pay hospitals in relation to Medicare is lower in LA than in San Diego, San Francisco, and Sacramento.

## Discussion

Dartmouth Medicare FFS data show that health care costs in LA are higher in comparison to other counties in California. However, RAND data indicate they are lower. In this analysis, we explored four explanations for why the ratio of what private insurers pay hospitals in relation to Medicare is lower in LA than San Diego, San Francisco, and Sacramento counties. Even though LA has the highest uninsured rate among these counties, private insurers are paying hospitals significantly more in San Diego, San Francisco, and Sacramento counties. This observation disconfirms the widely held belief that private insurers pay more in communities where a larger portion of the population is uninsured.

The Dartmouth Atlas Project is built on FFS claims data. Since MA may remove healthier enrollees, FFS is left with more expensive seniors. However, enrollment in MA was not higher in LA. When examining the counties by current health insurance coverage, LA had the highest percentage of individuals enrolled in Medicaid and the lowest percentage of individuals enrolled in Medicare. However, in LA, Medicare costs were lowest and Medi-Cal costs were highest.

Ultimately, none of these first three hypotheses explained the lower ratio of private to Medicare costs in LA. However, the HHI suggested that the LA market is more competitive. Although LA might be the most expensive county when hospitals do not compete on price, such as Medicare FFS, private insurers are better able to negotiate price when there is an oversupply of hospitals. These results are consistent findings in an unpublished white paper by Kronick and Neyaz that identified and ranked the largest and smallest private insurance to Medicare ratios among hospitals in California [17]. They reported four of the ten lowest ratios are in LA, while only one hospital in LA is among those with the top ten largest ratios [17]. These findings underscore the need for more research that includes private health care claims. Concentrating on the Medicare Fee for Service population, as in the Dartmouth Atlas Project data, may obscure the more complete picture that emerges from data representing the full range of payers.

### Limitations

Our results should be interpreted considering several important limitations. First, it was challenging to find public sources listing Medicare and Medicaid costs by county in California. As a result, we used data from two different sources, CHIS and KFF, to calculate these costs. We could only find average per capita Medicare and Medicaid spending at the state level, and we had to assume that this spending did not vary across counties. Through conversations with California health care leaders, we learned that counties independently negotiate Medicaid fees. As a result, spending by enrollee can vary drastically across counties. Unfortunately, these data are not publicly available. Without data access, it is difficult to determine how it would impact the results.

Interviews with another California expert suggested other limitations in the KFF and CHIS data sources. KFF estimates for California are based on state budget data that does not include significant funding sources such as expenditures for In-Home Support Services, Department for Developmental Services, or hospital programs that use public health expenditures. Although KFF includes federal funds, this gives the false impression that the state of California underestimates payments to public hospitals. Further, CHIS data do not reconcile with state administrative data. CHIS data significantly understate the Medi-Cal enrollment dates in comparison to the state of California’s enrollment data. Although using KFF and CHIS data to calculate Medicare and Medicaid costs by county has its limitations, no publicly available source provides this information. For these reasons, results from this study must be interpreted with caution.

A final limitation is that it is unclear whether the higher number of hospitals reflects oversupply. Unfortunately, we are unable to make this determination from the data we had available. Future research should investigate this issue in more detail.

## Conclusion

In summary, datasets based on Medicare do not provide an accurate representation of hospital spending for the general US population, but rather for the limited group of Medicare FFS enrollees. Although Dartmouth Atlas of Health Care data suggest that hospital costs are highest in LA, analysis of RAND data indicates that private insurers are paying hospitals less in relation to Medicare contracts in LA than San Diego, San Francisco, and Sacramento counties. The hypotheses investigated: differences in demographics, enrollment rates in MA, and the number of individuals enrolled in Medicare and Medi-Cal, failed to explain why LA hospital costs are lower for those not in Medicare FFS. However, the HHI indicates that high market competition among hospitals could explain this difference. Future research is required to understand this relationship. In addition, we need publicly available data to untangle the significant regional differences in California hospital costs.

## Data Availability

Data for this analysis came from several sources. To estimate hospital prices, we used data from self-insured employers, state-based all-payer claims databases, and health plans that have been assembled by RAND [11]. The hospital data were from 2016–2018. However, we believe these indicators remain relatively stable. Relative prices were estimated from the allowed amount paid by the private plan as a percentage of what Medicare pays (by pre-negotiated contract) the same hospital for the same services. The data can be found here: https://www.rand.org/pubs/research_reports/RR4394.html. Hospital price data were categorized by county group based on zip code. Statistical tests and descriptive analyses, and graphic displays were completed using R. The U.S. Census Bureau 2020 [12] (https://data.census.gov/cedsci/profile?g=0500000US06037) and the California Health Interview Survey (CHIS) [13] (http://healthpolicy.ucla.edu/CHIS/Pages/default.aspx) were used to better understand the demographic makeup of each county. Other cost data from Kaiser Family Foundation (KFF) [6] were used to calculate 2019 Medicaid and Medicare spending per enrollee (https://www.kff.org/other/state-indicator/total-population/?currentTimeframe=0&sortModel=%7B%22colId%22%3A%22Location%22%2C%22sort%22%3A%22asc%22%7D). Data from the California Department of Health Care Services were used to examine Medicare Advantage enrollment data by county [14] (https://www.dhcs.ca.gov/services/Documents/OMII-Medicare-Databook-February-18-2022.pdf). State- and county-level enrollment numbers are from August 2021. To examine how competitive the different health care markets are and the concentration of hospitals in each of the various markets, we used the Herfindahl–Hirschman Index (HHI) from the Yale Tobin Center for Economic Policy [15] (Rhoades SA. The Herfindahl–Hirschman Index. Fed. Res. Bull. 2019;79:188).
